# Haste makes waste: Early nutrition prescription for critically ill patients

**DOI:** 10.1002/agm2.12337

**Published:** 2024-06-14

**Authors:** Siying Chen, Zhigang Chang

**Affiliations:** ^1^ Department of Critical Care Medicine, Beijing Hospital, National Center of Gerontology, Institute of Geriatric Medicine Chinese Academy of Medical Sciences Beijing China; ^2^ Graduate School of Peking Union Medical College Beijing China

**Keywords:** critically ill, early nutrition, nutritional support

The Nutrirea‐3 study was an impactful, multicenter, open‐label parallel‐group randomized controlled trial (RCT) conducted in 61 intensive care units (ICUs) across France.^1^ The study included patients who were receiving invasive mechanical ventilation and vasopressor therapy for shock, with a median Sequential Organ Failure Assessment (SOFA) score of 10 (8–13) and a median Simplified Acute Physiology Score (SAPS) II of 60 (48–74 points, study group) and 61 (48–74 points, control group), respectively. In total, 3044 patients were randomized into two groups: the low‐calorie, low‐protein study group (6 kcal/kg/day, 0.2–0.4 g/kg/day) and the standard‐calorie, standard‐protein control group (25 kcal/kg/day, 1.3 g/kg/day). Enteral nutrition (EN) was administered continuously for 24 h at a constant rate to achieve caloric and protein targets on the first day. The primary outcomes were 90‐day all‐cause mortality and the number of days until the ICU discharge criteria were met. The secondary outcomes included the rates of secondary infections, gastrointestinal (GI) events, and liver dysfunction. There was no difference in 90‐day mortality between the two groups, indicating that high or low calorie and protein intake during the first week did not affect the 90‐day mortality rate. The patients in the study group met the ICU discharge criteria 1 day earlier than did those in the control group. Regarding the secondary outcomes, the low‐calorie feeding group exhibited lower rates of vomiting, diarrhea, intestinal ischemia, and liver dysfunction, suggesting that increased feeding may be harmful.

In the past, there was a notion that early and adequate nutrition could improve outcomes, where “adequate” meant achieving sufficient energy intake goals in the early stages of critical illness. However, the publication of the EPaNIC trial in 2011 led to skepticism about this concept.^2^ In this trial, patients who received early supplemental parenteral nutrition (PN) had adverse outcomes that were initially attributed to PN itself. This interpretation led to a significant reduction in the use of PN in clinical practice and recommendations against the use of PN in guidelines.^3^ Since then, accumulating evidence has suggested that providing complete energy targets (i.e., managing nutrition to cover estimated energy expenditure entirely) is detrimental rather than beneficial, regardless of the delivery route. This suggests that clinicians may overestimate patients' energy needs, overfeed patients, or, conversely, encounter biological benefits associated with underfeeding in patients. Some studies have evaluated potential biological mechanisms underlying previous unexpected findings, such as an increased endogenous energy supply independent of the exogenous energy supply and the nutritional suppression of autophagy.^4^

In the Nutrirea‐3 study, patients in the standard feeding group experienced a rapid increase in caloric intake on the first, second, and third days, far exceeding the recommended intake levels. Similarly, the protein intake in the standard feeding group increased significantly on the days leading up to ICU admission. Although the study did not show differences in mortality rates, early overfeeding and excessive protein intake in the days before ICU admission may have had adverse effects on the GI tract. Furthermore, the patient population in the Nutrirea‐3 study included individuals in shock who were receiving high‐dose vasopressors (with a median dose of 0.5 μg/kg/min in both groups). Targeting 100% of the energy goals for these patients in the early stages of critical illness is apparently uncommon, particularly for those receiving high‐dose vasopressors. Therefore, the continuous adoption of this approach should be avoided. In specific patient subgroups, the potential harms associated with high‐dose energy and protein intake underscore the importance of providing nutritional support to critically ill patients in a more personalized manner. Finally, while the nutritional strategies of maintaining intake levels at 6 and 25 kcal/kg/day were implemented by the study and control groups, respectively, in the Nutrirea‐3 study for the initial 7 days, which may have been applicable to the study itself, these results should not be generalized in clinical practice. Restricting the nutritional intake of all patients to 6 kcal/kg/day for 7 days, followed by a rapid increase to 30 kcal/kg/day, may pose a significant risk of refeeding syndrome. The difficulty in identifying refeeding syndrome when a rapid transition from low to high doses occurs concurrently with a patient's transfer from the ICU to a regular ward is particularly dangerous.

Patients with critical illnesses requiring organ support typically have a higher mortality rate, and among survivors, an extended recovery period is often necessary. The acute phase is considered a critical period characterized by organ dysfunction, anorexia, metabolic imbalances, endocrine disruptions, and severe catabolism, accompanied by significant muscle wasting^5^; nutritional support becomes paramount during this stage. Studies have demonstrated that more severe deficits in energy and protein intake are closely linked to higher rates of healthcare‐associated infections, ICU‐acquired weakness, prolonged durations of invasive mechanical ventilation, extended ICU stays, and increased mortality risk.^6^

Malnutrition is often associated with adverse outcomes, leading to the general belief that meeting full energy requirements is necessary.^7^ However, in critically ill patients, various metabolic changes may occur, and these changes vary from patient to patient. The range of metabolic rate changes is significant, with approximately 50% of patients experiencing an increase in their metabolic rate, while others experience a decrease in their metabolic rate. As energy sources shift from primarily fat oxidation to glucose oxidation, glycolysis increases.^8^ Protein hydrolysis accelerates, especially in cases of prolonged starvation and metabolic imbalance, without significant stimulation of ketogenesis or gluconeogenesis. An exogenous supply of fat or carbohydrates weakens the normal inhibition of fat breakdown and protein hydrolysis.^8^ Furthermore, excessive calorie availability can be harmful, particularly as it increases the production of oxygen free radicals, which are harmful to mitochondria.^9^ Complete EN may lead to GI intolerance, thereby heightening the risk of infection. On the other hand, limiting caloric intake may enhance autophagy, which may be a protective factor.^10^

In NUTRIREA‐2, a multicenter study, researchers enrolled 4000 mechanically ventilated patients, two‐thirds of whom were experiencing septic shock. During the initial critical illness phase, patients were randomized to receive either early full‐dose enteral or parenteral nutrition, with 28‐day mortality as the primary endpoint. No statistically significant difference in mortality was observed between the two groups. However, compared to those receiving parenteral nutrition, patients receiving enteral nutrition exhibited greater rates of adverse events such as vomiting (34% vs. 24%, *p* ≤ 0.0001), diarrhea (36% vs. 33%, *p* = 0.007), and clinical management for intestinal ischemia (2% vs. <1%, *p* = 0.007).^11^ Compared to full PN, full EN might pose a risk to shock patients, with rare but serious complications such as Ogilvie's syndrome and acute mesenteric ischemia, which still require attention.^11^, ^12^ Additional studies have provided additional evidence supporting the correlation between GI intolerance occurring during EN and increased mortality rates.^13^ In the NUTRIREA‐3 study, the feeding strategy was determined by bedside physicians, with 61.6% (low feeding) and 56.0% (standard feeding) of patients receiving full EN. Consequently, the NUTRIREA‐3 study showed lower rates of vomiting, diarrhea, intestinal ischemia, and liver dysfunction in the low‐calorie feeding group, which could be explained by secondary outcome measures.^1^

The pathophysiological characteristics of metabolism during early‐stage critical illness include inflammatory responses, increased energy expenditure, insulin resistance, and catabolic metabolic reactions, leading to the depletion of energy reserves such as hepatic glycogen (glucose), fat (fatty acids), and muscle (amino acids). As the disease gradually progresses, endogenous energy production decreases, a process that may persist until the acute phase of the illness subsides.^14^ Consequently, critically ill patients generate a substantial amount of endogenous energy during the acute phase, which may result in caloric and protein deficits. Therefore, feeding ICU patients differs significantly from feeding healthy individuals. Stress or inflammatory responses can act as barriers to nutrient absorption and utilization, and early adequate nutritional provision may increase the patient burden and hinder recovery. A prospective observational study encompassing 626 mechanically ventilated patients with circulatory shock from 14 countries investigated the effects of early EN support (EEN group, ICU admission <48 h) compared to delayed enteral nutrition support (DEN group, ICU admission >48 h). The findings revealed a lower incidence of persistent organ dysfunction and mortality (*p* = 0.04) in the EEN group. Furthermore, patients in the EEN group exhibited prolonged survival times and a reduced duration of vasopressor use.^15^ Hence, we recommend initiating EN promptly. However, rapidly escalating caloric and protein intake within 24–48 h of critical illness onset to attain full feeding goals is detrimental, conflicting with the stepwise approach emphasized by the ESPEN guidelines.^16^ We advocate early trophic feeding, administering 10–20 kcal/kg/day or no more than 500 kcal/day, to mitigate GI intolerance. Gradually reaching 80% of the target intake within 1 week is deemed the optimal strategy.^3^

Muscles serve as the primary source of endogenous amino acids, and muscle mass upon ICU admission is positively correlated with the prognosis of critically ill patients.^17^ For multiple organ dysfunction syndrome (MODS) patients, the breakdown of the metabolic response in the first 10 days after ICU admission can lead to a reduction in muscle mass of up to 1 kg/day.^5^ Mechanistic studies have shown that providing high‐dose protein has beneficial effects on muscle mass loss and muscle protein synthesis.^18^ Some studies have suggested that supplementing adequate protein may improve the prognosis of critically ill patients.^19^ However, we need to carefully consider the timing and dosage of protein administration.

Post hoc analysis of the EPaNIC study comparing early and late supplemental PN revealed an association between early high protein intake and adverse patient outcomes.^20^ This was also confirmed in another retrospective study, the PROTINVENT study, where patients with a protein intake <0.8 g/kg/day had the highest 6‐month mortality rates, and those with a high protein intake (>0.8 g/kg/day) in the first 3 days also had increased mortality rates.^21^ A recent RCT (the EFFORT Protein Trial) enrolled 1329 patients from 85 ICUs across 16 countries. Within 96 h of ICU admission, patients commenced supplementation with either high‐dose protein (≥2.2 g/kg/day) or standard‐dose protein (≤1.2 g/kg/day), continuing until day 28, death, or transition to oral feeding.^22^ The results showed that providing larger doses of protein did not improve survival to discharge in critically ill patients with acute kidney injury and high organ failure scores and may lead to adverse outcomes.

It is generally believed that proteins and nutrients can inhibit autophagy, an essential cellular clearance mechanism. However, controversy remains regarding whether this approach can prevent inadequate autophagy.^18^ A recent retrospective study revealed no adverse effects of early protein supplementation during ICU admission but showed improved 60‐day survival rates. In this study, moderate amounts of protein were administered during the first 3 days.^23^ Based on this limited information and the principle of primum non nocere, gradually increasing the protein target is recommended. This approach also applies to calorie intake, with gradual increases in EN targets over several days. According to the ESPEN guidelines, the protein target should be gradually increased to at least 1.3 g/kg/day.^24^

The NUTRIREA‐3 study delves into the optimal caloric and protein intake for a specific subset of critically ill patients with circulatory shock receiving enteral nutrition. It affirms that early initiation of full‐dose EN during the acute phase of critical illness is detrimental for severely ill patients requiring high doses of vasopressor agents. EN support should prioritize early initiation but with a “less is more” approach. Despite recent large multicenter RCTs shedding light on the role, timing, and dosage of early EN in septic shock, the heterogeneity of ICU patients with diverse nutritional needs, tolerances, and disease severities impedes the establishment of a universal nutritional therapy protocol. Hence, a one‐size‐fits‐all nutritional strategy is not feasible for ICU patients. Instead, decisions regarding EN should be tailored to each patient's individual benefits, risks, and tolerability.

## AUTHOR CONTRIBUTIONS

Ms. Siying Chen and Dr. Zhigang Chang conceptualized the manuscript. Ms. Siying Chen and Mr. Zhigang Chang did the literature search and wrote the manuscript draft. Dr. Zhigang Chang critically revised the manuscript draft.

## FUNDING INFORMATION

This work was not supported by any source.

## CONFLICT OF INTEREST STATEMENT

This work was not supported by any source.
